# A real-world analysis of trametinib in combination with hydroxychloroquine or CDK4/6 inhibitor as third- or later-line therapy in metastatic pancreatic adenocarcinoma

**DOI:** 10.1186/s12885-023-11464-3

**Published:** 2023-10-10

**Authors:** Hui Tang, Yuping Ge, Tingting You, Xiaoyuan Li, Yingyi Wang, Yuejuan Cheng, Chunmei Bai

**Affiliations:** Department of Medical Oncology, Peking Union Medical College Hospital, Chinese Academy of Medical Sciences & Peking Union Medical College, Beijing, China

**Keywords:** Pancreatic cancer, Later-line therapy, MEK inhibitor, Autophagy, Real-life data

## Abstract

**Background:**

There are no standard third-line treatment options for metastatic pancreatic ductal adenocarcinoma (mPDAC). Trametinib in combination with hydroxychloroquine (HCQ) or CDK4/6 inhibitors for pancreatic adenocarcinoma showed promising efficacy in preclinical studies. However, the regimens have not been well examined in patients with mPDAC.

**Methods:**

Patients with mPDAC who received the combination of trametinib and HCQ or CDK4/6 inhibitors as third- or later-line therapy were reviewed. The efficacy and prognosis were further analyzed.

**Results:**

A total of 13 mPDAC patients were enrolled, of whom 8 and 5 patients were treated with trametinib plus HCQ or a CDK4/6 inhibitor (palbociclib or abemaciclib), respectively. All enrolled patients had either KRAS G12D or G12V mutations and had received a median of 3 prior lines of therapy (range, 2–6). The median trametinib treatment duration was 1.4 months. Of the 10 patients with measurable disease, only 1 patient achieved stable disease, and the remaining patients had progressive disease. Moreover, in patients treated with trametinib plus HCQ and a CDK4/6 inhibitor, the median progression-free survival was 2.0 and 2.8 months, respectively, and the median overall survival was 4.2 and 4.7 months, respectively. Moreover, 5 (50%) patients experienced grade 3–4 adverse events in 10 patients with available safety data.

**Conclusions:**

The combination of trametinib and HCQ or CDK4/6 inhibitors may not be an effective later-line treatment for mPDAC, and the current preliminary findings need to be confirmed by other studies with larger sample sizes.

## Introduction

Pancreatic cancer is a common malignancy and has become the third leading cause of cancer-related death [1, 2]. More than 80% of patients with pancreatic ductal adenocarcinoma (PDAC) have locally advanced or metastatic disease at the time of diagnosis, and the 5-year survival rate is only about 12% [2]. Systemic chemotherapy has been considered the main treatment strategy for metastatic PDAC (mPDAC). Although the newly developed FOLFIRINOX (combination chemotherapy with fluorouracil, leucovorin, irinotecan, and oxaliplatin) and GnP (gemcitabine plus nab-paclitaxel) regimens have improved the survival outcome of patients with mPDAC in the past decade, these regimens generally can only restrain tumor progression for approximately half a year [3, 4]. However, after receiving FOLFIRINOX and GnP regimens and disease progression, patients with mPDAC have few effective subsequent therapeutic options, which also contributes to their poor prognosis [5, 6].

Kirsten Rat Sarcoma (KRAS) is the most commonly mutated oncogene in pancreatic cancer, found in approximately 90% of PDAC cases, and can regulate pathways involved in tumor cell survival and proliferation [6]. Unfortunately, KRAS was considered undruggable until the recent breakthrough in therapies targeting mutant KRAS G12C [7]. Inhibitors targeting other KRAS oncogene mutations, such as G12D and G12V, are still mainly in phase I clinical trials (ClinicalTrials.gov NCT05737706, NCT05533463, and NCT05846516). Hence, it becomes a reasonable choice to target downstream signaling pathways that are aberrantly activated due to mutant KRAS, including mitogen-activated protein kinase kinase (MEK) [6]. Preclinical studies have proven that basal autophagy is very active in KRAS-mutant PDAC cells, while trametinib (a MEK inhibitor) plus hydroxychloroquine (HCQ, an autophagy inhibitor) showed encouraging synergistic cytotoxic effects in PDAC animal models [8]. On the other hand, as important regulatory elements of cell proliferation, cyclin-dependent kinases (CDKs) were reported to induce metabolic reprogramming and vulnerabilities in PDAC [9] and have a synthetic lethal interaction with KRAS [10]. The combination of trametinib and CDK4/6 inhibitors in KRAS-mutated solid tumors, including PDAC, lung cancer, and colorectal cancer, also elicited a robust therapeutic response [10–12]. However, there were only sporadic case reports of 4 and 6 PDAC patients receiving trametinib in combination with HCQ or CDK4/6 inhibitors, respectively, raising concerns about publication bias [8, 11, 13, 14].

Herein, we report our single-center data on trametinib in combination with HCQ or CDK4/6 inhibitors in the treatment of patients with mPDAC.

## Materials and methods

### Patients

Patients with PDAC who received trametinib in combination with HCQ or CDK4/6 inhibitors as third- or later-line therapy at Peking Union Medical College Hospital (PUMCH) between June 2020 and December 2022 were reviewed using the Electronic Medical Record Analytical Database (PUMCH-EMERALD). The inclusion criteria were as follows: (1) patients with histologically confirmed mPDAC and documented somatic KRAS point mutations; (2) age 18 years or older; and (3) received trametinib plus HCQ or CDK4/6 inhibitors as third- or later-line therapy. The exclusion criteria were as follows: (1) patients who died or were lost to follow-up within one month after trametinib initiation; (2) combined with other primary tumors; and (3) survival outcomes could not be assessed. Data retrieved from medical records and telephone follow-ups were analyzed to evaluate efficacy and toxicity. Informed consent was not required due to the anonymization of the data and the retrospective design.

### Treatment and assessment

All patients treated with trametinib fell into the following lab parameters: favorable renal, liver, and bone marrow function (serum creatinine concentration ≤ 1.5 mg/dl; total serum bilirubin concentration ≤ 3 mg/dl; leucocyte count ≥ 3000 cells/μL; hemoglobin concentration ≥ 8 g/dL; platelet count ≥ 100000/μL). For patients with KRAS mutations, clinicians administered the appropriate dose of trametinib (1–2 mg once daily) and HCQ (200–600 mg once or twice daily) according to the patient's performance status and tolerance. In particular, if coalterations in cyclin pathway genes occurred, the patient was treated with a CDK4/6 inhibitor (palbociclib 50–75 mg once daily for 21 days followed by 7 days off or abemaciclib 100–200 mg once or twice daily) rather than HCQ. For patients with an ECOG score of 0–1, clinicians may choose the highest of these doses, while for patients with an ECOG score of 2, clinicians would use the lowest dose as the starting dose and gradually increase the dose to the highest, depending on the toxicities. Treatments continued until intolerant toxicity, disease progression, or death.

All patients were investigated by computed tomography (CT) scans and/or magnetic resonance imaging (MRI) every 1 to 2 months. Treatment responses were categorized as progressive disease (PD), stable disease (SD), partial response (PR), and complete response (CR) based on the Response Evaluation Criteria in Solid Tumors version 1.1 [15]. Carbohydrate antigen 19–9 (CA19-9) was measured within one month before treatment onset and every 1–2 months thereafter. Overall survival (OS) was defined as the time from the initiation of trametinib until death from any cause. Progression-free survival (PFS) was defined as the time from trametinib onset to disease progression or death due to any cause. Treatment-related adverse event (AE) severity was graded according to the Common Terminology Criteria for Adverse Events version 5.0. All patients were followed up until death or loss of contact, with a follow-up deadline of May 2023.

### Statistical analysis

All statistical analyses were conducted using R software (version 3.6.1, https://www.r-project.org/). Survival outcome was evaluated by Kaplan–Meier analyses. Cox proportional hazard models were used to calculate the hazard ratios (HRs) with 95% confidence intervals (CIs) of variables associated with survival outcomes in patients. A two tailed probability value of *p* < 0.05 was considered statistically significant.

## Result

### Patient characteristics

A total of 13 patients with mPDAC were enrolled (Table 1), of whom 8 and 5 patients were treated with trametinib plus HCQ and CDK4/6 inhibitors (3 with palbociclib and 2 with abemaciclib), respectively. The median age was 60 years (range, 30–77), 9 (69.2%) of the patients were male, and 4 (30.8%) and 9 (69.2%) of the patients had ECOG performance status scores of 0–1 and 2, respectively. Patients had received a median of 3 prior systemic antitumor regimens (range, 2–6). Moreover, all enrolled patients harbored either KRAS G12D (*n* = 6) or G12V (*n* = 7) mutations, and 5 had CDKN2A (*n* = 4) or CDKN1B (*n* = 1) mutations.

**Table 1:** Clinical profile of all enrolled patients

	**Total**	**Hydroxychloroquine**	**CDK4/6 inhibitor**
	**(*n* = 13)**	**(*n* = 8)**	**(*n* = 5)**
Age, median (range), years	60 (50, 77)	58.5 (50, 77)	60 (51, 72)
Sex, male	9 (69.2%)	6 (75.0%)	3 (60.0%)
Smoking history, yes	3 (23.1%)	2 (25.0%)	1 (20.0%)
Drinking history, yes	4 (30.8%)	3 (37.5%)	1 (20.0%)
Diabetes, yes	3 (23.1%)	1 (12.5%)	2 (40.0%)
Performance status			
0–1	4 (30.8%)	4 (50.0%)	0 (0%)
2	9 (69.2%)	4 (50.0%)	5 (100%)
CA19-9, median (range), U/ml	840 (1, 20500)	1290 (13, 9480)	65 (1, 20500)
TNM stage IV	13 (100%)	8 (100%)	5 (100%)
Liver metastasis	11 (84.6%)	7 (87.5%)	4 (80.0%)
Multiple metastases	5 (38.5%)	2 (25.0%)	3 (60.0%)
Line of therapy			
3	4 (30.8%)	3 (37.5%)	1 (20.0%)
4	5 (38.5%)	2 (25.0%)	3 (60.0%)
5–7	4 (30.8%)	3 (37.5%)	1 (20.0%)
KRAS mutation			
G12D	6 (46.2%)	4 (50.0%)	2 (40.0%)
G12V	7 (53.8%)	4 (50.0%)	3 (60.0%)
Cyclin gene mutation			
CDKN2A	4 (30.8%)	0 (0%)	4 (80.0%)
CDKN1B	1 (7.7%)	0 (0%)	1 (20.0%)
None	8 (61.5%)	8 (100%)	0 (0%)

### Treatment outcome

By the time of the last follow-up, all patients had stopped trametinib treatment, and the median trametinib treatment duration was 1.4 months. Treatment discontinuations were caused by disease progression, treatment-related adverse events (AEs), and tumor-related AEs (e.g. bowel obstruction) in 5, 3, and 5 patients, respectively (Table 2). Toxicity data were not obtained in 3 patients because detailed medical records were not available and AEs could not be assessed via telephone follow-up. Among the 10 patients who were evaluable for treatment safety, 5 (50%) patients experienced grade 3–4 AEs, and the most common grade 3–4 AEs were nausea (*n* = 3) and fatigue (*n* = 2).

**Table 2:** Summary of treatment outcomes

	**Total**	**Hydroxychloroquine**	**CDK4/6 inhibitor**
	**(*n* = 13)**	**(*n* = 8)**	**(*n* = 5)**
Reason for treatment discontinuation			
Disease progression	5 (38.5%)	2 (25.0%)	3 (50.0%)
Tumor-related AEs	5 (38.5%)	4 (50.0%)	1 (20.0%)
Treatment-related AEs	3 (23.1%)	2 (25.0%)	1 (20.0%)
Change in CA19-9			
Declined over 30%	3 (23.1%)	1 (12.5%)	2 (40.0%)
Declined less than 30%	4 (30.8%)	4 (50.0%)	0 (0%)
Not expressed	3 (23.1%)	1 (12.5%)	2 (40.0%)
Unknown	3 (23.1%)	2 (25.0%)	1 (20.0%)
Treatment response			
Progressive disease	9 (69.2%)	5 (62.5%)	4 (80.0%)
Stable disease	1 (7.7%)	1 (12.5%)	0 (0%)
Unknown	3 (23.1%)	2 (25.0%)	1 (20.0%)

*Abbreviation*: *AEs* adverse events

As shown in Table 2, of the 10 patients with measurable disease (6 treated with trametinib plus HCQ, 4 treated with trametinib plus CDK4/6 inhibitors), only 1 patient who was treated with trametinib plus HCQ achieved SD, and the remaining patients had PD. Of the 7 patients with elevated CA19-9 at baseline and available CA19-9 data throughout trametinib treatment, no patient achieved the best reduction to normal CA19-9 levels, while 3 patients had a > 30% reduction from the pretreatment level.

### Survival outcome

**Fig. 1:**
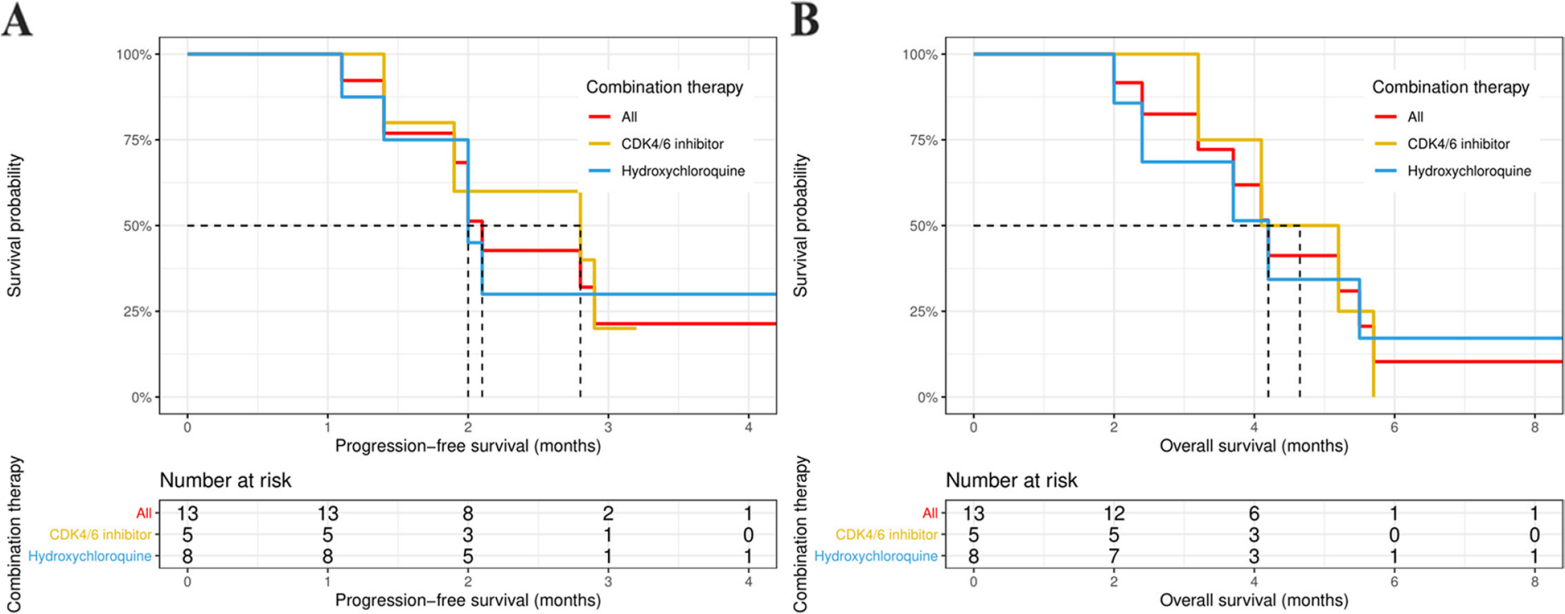
Kaplan–Meier curves of disease-free survival (**A**) and overall survival (**B**) in patients treated with trametinib in combination with hydroxychloroquine or CDK4/6 inhibitor

The median follow-up time was 3.7 months (range, 1.5–23.1). Up to the last follow-up, 9 of the 13 patients had disease progression, and 10 patients died. The median PFS was 2.1 months (95% CI: 1.9 months, not reached [NR]), and the median OS was 4.2 months (95% CI: 3.2 months, NR) in the whole cohort (Fig. 1). Specifically, in patients treated with trametinib plus HCQ or a CDK4/6 inhibitor, the median PFS was 2.0 and 2.8 months (95% CI: 2.0 months, NR; and 1.9 months, NR), respectively, and the median OS was 4.2 and 4.7 months (95% CI: 2.4 months, NR; and 3.2 months, NR), respectively.

To preliminarily explore the factors associated with the survival outcome, Cox regression analyses were performed. As shown in Table 3, univariate and multivariate analyses only confirmed that elevated pretreatment CA19-9 was an independent indicator of poor PFS (HR 13.54, 95% CI: 1.36, 134.66, *p* = 0.026), while none of the variables was significantly associated with OS (Table 4).

**Table 3:** Univariate and multivariate analyses of progression-free survival

Variables	Univariate analysis		Multivariate analysis	
	**HR (95% CI)**	***p***	**HR (95% CI)**	***p***
Gender (Female vs. Male)	0.79 (0.20,3.21)	0.743	-	-
Age (Years)	0.97 (0.87,1.08)	0.586	-	-
Smoking history (Yes vs. No)	0.28 (0.04,2.28)	0.235	-	-
Drinking history (Yes vs. No)	0.63 (0.13,3.03)	0.561	-	-
Diabetes (Yes vs. No)	0.59 (0.11,3.08)	0.527	-	-
Liver metastases (Yes vs. No)	0.75 (0.15,3.75)	0.727	-	-
Multiple metastases (Yes vs. No)	2.05 (0.55,7.70)	0.287	-	-
Line of therapy (4 vs. 3)	1.29 (0.23,7.13)	0.772	-	-
Line of therapy (5–7 vs. 3)	1.57 (0.25,9.76)	0.628	-	-
KRAS mutation (G12V vs. G12D)	0.29 (0.07,1.25)	0.097	-	-
Performance status (2 vs. 0–1)	0.46 (0.10,2.08)	0.313	-	-
Elevated CA19-9 (Yes vs. No)	13.54 (1.36,134.66)	**0.026**	13.54 (1.36,134.66)	**0.026**
Combination therapy (Hydroxychloroquine vs. CDK4/6 inhibitor)	1.20 (0.31,4.66)	0.796	-	-

**Table 4:** Univariate and multivariate analyses of overall survival

Variables	Univariate analysis		Multivariate analysis	
	**HR (95% CI)**	***p***	**HR (95% CI)**	***p***
Gender (Female vs. Male)	0.54 (0.11,2.69)	0.454	-	-
Age (Years)	0.93 (0.82,1.05)	0.248	-	-
Smoking history (Yes vs. No)	1.97 (0.46,8.39)	0.361	-	-
Drinking history (Yes vs. No)	3.16 (0.77,12.96)	0.110	-	-
Diabetes (Yes vs. No)	1.50 (0.36,6.24)	0.580	-	-
Liver metastases (Yes vs. No)	4.18 (0.51,34.39)	0.183	-	-
Multiple metastases (Yes vs. No)	0.74 (0.19,2.87)	0.662	-	-
Line of therapy (4 vs. 3)	3.50 (0.39,31.71)	0.265	-	-
Line of therapy (5–7 vs. 3)	4.56 (0.48,43.31)	0.186	-	-
KRAS mutation (G12V vs. G12D)	1.66 (0.43,6.40)	0.464	-	-
Performance status (2 vs. 0–1)	3.09 (0.38,25.46)	0.294	-	-
Elevated CA19-9 (Yes vs. No)	0.50 (0.08,3.01)	0.447	-	-
Combination therapy (Hydroxychloroquine vs. CDK4/6 inhibitor)	1.04 (0.28,3.89)	0.958	-	-

### Representative cases

**Fig. 2:**
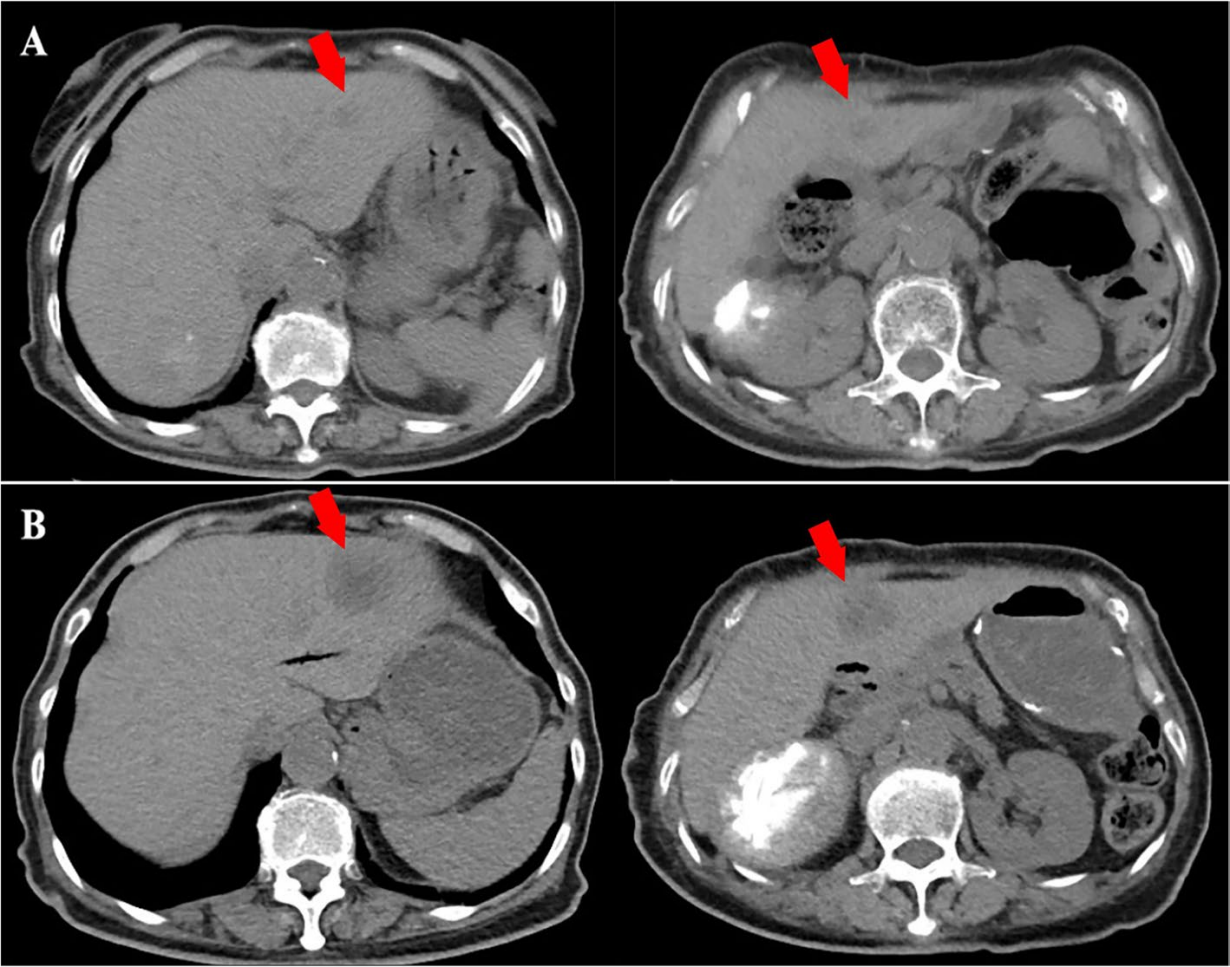
Computed tomography (CT) findings. **A** Plain CT scan obtained before initiation of treatment with trametinib plus hydroxychloroquine. **B** Plain CT scan obtained after 8 weeks of treatment shows enlarging hepatic metastatic lesions (arrows)

Ms. Xu was a 77-year-old female, who was diagnosed with mPDAC in January 2022 and had KRAS G12V, TP53, and APC mutations. She was treated with GS (gemcitabine plus S-1) and GnP regimens as first-, and second-line therapy, and the PFS was 22 and 12 weeks, respectively. Then, she received trametinib (1 mg once daily) in combination with HCQ (200 mg once daily). After 8 weeks of treatment, CT scans revealed that the number and diameter of liver metastases increased (Fig. 2). She was lost to follow-up after her last radiographic evaluation.

Ms. Qian was a 72-year-old female, who was initially diagnosed in August 2018, and had KRAS G12D, TP53, and CDKN2A mutations. She was treated with GS (gemcitabine plus S-1), GnP, nab-paclitaxel plus S-1, and capecitabine plus irinotecan regimens as first- to fourth-line therapy. She was administered trametinib (1 mg once daily) in combination with palbociclib (75 mg once daily) in March 2021 but discontinued the treatment in May 2021 because of intolerable toxicity (stomatitis, fatigue, and fevers of unknown cause). During the treatment, she declined all imaging examinations but had PD on positron emission tomography-computed tomography in June 2021. She died of septic shock 3 months after discontinuation of the treatment.

## Discussion

Pancreatic cancer is currently the fourth leading cause of cancer-related death and is expected to be the second leading cause by 2030 [16]. Despite modest progress in chemotherapy, later-line treatment options for pancreatic cancer are still very limited. In addition to chemotherapy, immune checkpoint inhibitors and targeted therapy are also worthy of development as palliative treatment options for pancreatic cancer [17].

The RAS/RAF/MEK/ERK or mitogen-activated protein kinase (MAPK) pathway is a critical pathway involved in uncontrolled cell proliferation. Inhibiting the MAPK pathway by targeting MEK is an attractive strategy in the treatment of patients with malignancies, particularly those harboring KRAS mutations, which have long been considered undruggable but occur in approximately 90% of PDAC cases. Trametinib is an oral, highly selective, reversible allosteric MEK1/2 inhibitor [18]. Preclinical studies have shown that trametinib can inhibit phosphorylated extracellular-signal regulated kinase (ERK), which correlates with G1 cell cycle arrest, decreased cell proliferation, and induction of apoptosis [19]. In the first-in-human trial of trametinib monotherapy, 2 of the 26 PDAC patients who had received an unlimited number of previous treatments achieved PR [20]. Nevertheless, inhibition of the RAS/RAF/MEK/ERK pathway induces autophagy, a cellular process that recycles waste and protects PDAC cells from the cytotoxic effects induced by MAPK pathway inhibition [13, 21]. Therefore, the combination of MEK or ERK inhibitors and HCQ, an autophagy inhibitor, may yield better antitumor effects [21]. Remarkably, two independent groups have confirmed that chloroquine or HCQ monotherapy showed limited activity, but MEK inhibitors synergistically reinforced growth inhibition induced by HCQ in PDAC cells, organoids, and xenograft models [8, 21].

On the other hand, alterations in CDKN2A/1B and amplification of CDK4/6 can contribute to higher expression of CDK4/6, which can be targeted with CDK4/6 inhibitors [11]. Notably, alterations in cell cycle-associated genes occurred in approximately 31% of RAS-mutant cancers [22]. Compared with patients with only one of these pathways altered, survival was worse in cancer patients harboring alterations in both RAS and cell cycle–associated genes [22]. Consistently, Zhou et al. [10] found that the combined inhibition of CDK4/6 and MEK was effective in preclinical models of lung cancer with KRAS and CDKN2A mutations.

Although preclinical studies have repeatedly reported the activity of trametinib in combination with HCQ or CDK4/6 inhibitors in KRAS-driven tumors, only a few case studies have confirmed the efficacy of these two combined treatment regimens in patients with PDAC [8, 11, 13, 14], and the inherent publication bias of case reports cannot be excluded. Interestingly, Mehdi et al. [23] reported that 10 patients with PDAC received MEK inhibitors (trametinib or cobimetinib) combined with HCQ. The results showed that the objective response rate and disease control rate for this combination were 12% and 62.5%, respectively, and the median PFS and OS were 5.7 and 6.6 months, respectively. However, half of the patients in this study received MEK inhibitors combined with HCQ as either first- or second-line therapy, which cannot represent a routine for this combination, and the small sample sizes in the previous studies require further validation. Herein, we report our single-center data on the combination of trametinib and HCQ or CDK4/6 inhibitors in mPDAC patients with KRAS mutations. In contrast, our results demonstrate that the combination of trametinib and HCQ or CDK4/6 inhibitors could hardly control the progression of heavily treated PDAC. The disease control rate was as low as 10%, and the median PFS was approximately 2 months, which was almost the same as the imaging examination interval, suggesting that the combination of trametinib and HCQ or CDK4/6 inhibitors is not an effective regimen for mPDAC. There are many possible reasons contributing to trametinib resistance. First, the number of prior treatment lines and the patient's performance status undoubtedly affect the efficacy of trametinib [24], and in our cohort, approximately 70% of the patients had a performance status score of 2, receiving a median of 3 prior lines of therapy. Second, given the performance status and tolerability, patients were generally not treated with an adequate dose of trametinib in combination with HCQ or CDK4/6 inhibitors, and 8 patients discontinued trametinib early for reasons other than disease progression. Naturally, low-dose regimens also affect the clinical activity of the drugs, but such doses may be more practical in the context of later-line therapy. Third, overactivation of alternative pathways (e.g., PI3K/Akt) may contribute to MEK inhibitor primary and acquired resistance [25]. Furthermore, a randomized controlled trial found that the addition of trametinib to gemcitabine did not improve the treatment response and survival outcome in patients with mPDAC [26], suggesting that trametinib may not be as effective as expected in mPDAC.

To the best of our knowledge, this is the first cohort study to report the poor efficacy of the combination of trametinib and HCQ or CDK4/6 inhibitors in pancreatic cancer. There are several limitations in our research. First, the single-center approach may restrict the universality of our results to other circumstances. Second, the small sample size affects the reliability of the conclusions, although this is the largest study of trametinib in combination with HCQ or CDK4/6 inhibitors in pancreatic cancer. Our findings still need to be confirmed in larger studies, and an ongoing prospective study (ClinicalTrials.gov NCT03825289) to assess trametinib in combination with HCQ in patients with advanced PDAC would reach more instructive conclusions about the existing contradictions. Notably, our study suggests that the ongoing study on these combinations should continue with caution, and further clinical trials exploring these combinations in unselected patients with PDAC may not be justified.

## Conclusion

In summary, our results suggested that the combination of trametinib and HCQ or CDK4/6 inhibitors may not be an effective later-line treatment for patients with mPDAC, and the current preliminary findings need to be confirmed by other studies with larger sample sizes.

## Data Availability

All inquiries can be directed to the corresponding authors.
